# A novel function for spumaretrovirus integrase: an early requirement for integrase-mediated cleavage of 2 LTR circles

**DOI:** 10.1186/1742-4690-2-31

**Published:** 2005-05-18

**Authors:** Olivier Delelis, Caroline Petit, Herve Leh, Gladys Mbemba, Jean-François Mouscadet, Pierre Sonigo

**Affiliations:** 1 Génétique des virus, Département des Maladies Infectieuses, Institut Cochin, INSERM U567, CNRS UMR8104, Université René Descartes, 22 rue Méchain, 75014 Paris, France; 2 Bioalliancepharma, 59 boulevard Martial Valin, 75015 Paris, France; 3 LBPA, CNRS UMR8113, Ecole Normale Supérieure de Cachan, 61 avenue du Président Wilson, 94235, Cachan, France

**Keywords:** spumaretrovirus, integrase substrate, palindrome at LTR-LTR junctions, 2-LTR circles DNA

## Abstract

Retroviral integration is central to viral persistence and pathogenesis, cancer as well as host genome evolution. However, it is unclear why integration appears essential for retrovirus production, especially given the abundance and transcriptional potential of non-integrated viral genomes. The involvement of retroviral endonuclease, also called integrase (IN), in replication steps apart from integration has been proposed, but is usually considered to be accessory. We observe here that integration of a retrovirus from the spumavirus family depends mainly on the quantity of viral DNA produced. Moreover, we found that IN directly participates to linear DNA production from 2-LTR circles by specifically cleaving the conserved palindromic sequence found at LTR-LTR junctions. These results challenge the prevailing view that integrase essential function is to catalyze retroviral DNA integration. Integrase activity upstream of this step, by controlling linear DNA production, is sufficient to explain the absolute requirement for this enzyme.

The novel role of IN over 2-LTR circle junctions accounts for the pleiotropic effects observed in cells infected with IN mutants. It may explain why 1) 2-LTR circles accumulate *in vivo *in mutants carrying a defective IN while their linear and integrated DNA pools decrease; 2) why both LTRs are processed in a concerted manner. It also resolves the original puzzle concerning the integration of spumaretroviruses. More generally, it suggests to reassess 2-LTR circles as functional intermediates in the retrovirus cycle and to reconsider the idea that formation of the integrated provirus is an essential step of retrovirus production.

## Background

Integration of viral genomes into host cell DNA is a key element of the life cycle and pathogenesis of many viruses. DNA viruses integrate by relying solely on cell machinery. In contrast, retroviruses possess a specialized endonuclease, also designated integrase (IN), which is essential for their replication (for a review, see [1]). After entering a target cell, reverse transcriptase (RT) converts genomic RNA into linear double-stranded cDNA with a copy of the viral long terminal repeat (LTR) at each end. Such linear genomic cDNA included in a preintegration complex (PIC) [2-9] can be used as a template for integration *in vivo*. Consequently, circular viral genomes that are detected in infected cells were considered until now as «dead-end» molecules, without essential function in the integration process and the viral cycle in general [8].

Integration mediated by the retrovirus IN occurs in two catalytic steps, referred to as 3'-processing and strand transfer (or joining), respectively. Interestingly, the two steps appeared on distinct reactions catalyzed by virus IN in two different compartments in the infected cells. The strand transfer reaction joins viral DNA to cellular DNA in the cell nucleus. The viral cDNA ends are used to cut the target DNA in a staggered manner, which covalently links the viral 3' ends to the 5' phosphates of the cut (for reviews see [10,11]. The 3' hydroxyl groups at the LTR termini are the nucleophiles that promote DNA strand transfer [12]. Efficient strand transfer requires previous endonucleolysis of DNA that produces recessed 3'hydroxyl ends [3,5]. This occurs in the cytoplasm very soon after reverse transcription is completed [13-16], as viral genomes with blunt ends are extremely rare in the infected cytoplasm. Following these reactions, host cell enzymes likely repair the gap remaining between host and provirus DNA [17,18].

IN recognizes and acts on short sequences (12 to 20 bp) called attachment (*att*) sites that are located at the LTRs [19]. *Att *site includes the invariant CA dinucleotides, which are conserved in all retroviruses whereas the other nucleotides of the *att *site, while not conserved in sequence, form an (imperfect) inverted repeat (IR) in all retroviruses, that has to be maintained intact for viral replication. *Att *mutagenesis experiments showed that mutation in one LTR precludes the processing of the other, demonstrating that activity of IN is concerted onto the two viral LTRs that are simultaneously cleaved *in vivo *[20]. The structural basis of such concerted processing of both extremities is unknown. More surprisingly, in the case of spumaretroviruses, a subfamily of retroviruses that share some features of DNA viruses [21-23], the IN may process only one of the two LTRs, although the *att *sites are present at the two LTRs. Based on the sequences of both 2-LTR DNA and integrated proviruses, an asymmetric processing of *att *sites has been proposed, in which IN may cleave the right, U5 end and may leave the left, U3 end intact [24,25]. As the human spumaretrovirus (PFV) IN presents the usual features of other IN and carries out *in vitro *an endonucleolytic activity, as well as strand transfer and disintegrase activities [26,27], the reason for this unusual mechanics is not understood at present.

The *att *recognition site of IN is present at least one time on all forms of viral DNA. In addition to linear and integrated forms, viral DNA is found in the infected cells as covalently closed DNA circles containing either one or two copies of the LTR, referred to as 1-LTR and 2-LTR circles, respectively [2]. Interestingly in the 2-LTR circles, the *att *sites are in a closed configuration due to the juxtaposition of the two LTRs and are included within a palindromic motif formed by the inverted repeat sequences in all retroviruses [28-31]. These 2-LTR circles are believed to result from a direct covalent joining of LTR ends at the so-called circle junction [32,33]. Circularization is thought to occur by blunt-end ligation of the ends of linear proviral DNA, even no direct evidence has been provided until now to support this hypothesis. 2-LTR could be formed in part by the non-homologous end-joining (NHEJ) pathway of DNA recombination [34]. The two-LTR circle forms could, theoretically, serve as a potential precursor for the integrated provirus [4]. In spleen necrosis virus (SNV), Rous sarcoma virus (RSV), avian sarcoma virus (ASV) and avian leukosis virus (ALV), closed circular forms were initially proposed to act as substrates templates for integration [31,32,35], although these reports have not been substantiated. Although they are currently described in a productive infection as "dead end" molecules, precisely because of their incapacity to be directly integrated [8], intriguing observations invite some to reconsider their place. First, 2-LTR molecules were shown to be used as functional templates for the transcription machinery in HIV infected cells [36-39]. Second, 2-LTR viral DNA were detected in the cytoplasm of MLV and PFV infected cells at a very early time post infection, suggesting that they are not formed in the nucleus by an alternative fate to the integration way [40,41]. In this context, we asked whether 2-LTR circles, rather than being substrate for integration nor "dead end" molecules, would be used as substrates for a preintegrative endonucleolytic activity of PFV IN.

Such interrogation comes within the scope of the more global questioning concerning the pleiotropic actions of IN. Indeed, the mechanisms underlying the essential requirement for integration are still unclear in the retrovirus cycle. Why is integration critical for viral production when unintegrated DNA is abundant and competent for transcription [36-39,42-45]? Is it possible that preintegrative function of IN explain its essential requirement rather than integration *per se*? Indeed, in addition to its roles in the establishment of the proviral integrated state, IN participates to other critical steps, such as reverse transcription [23,46-52], nuclear import of HIV-1 preintegration complex (PICs) (for a review, see [53]), and the postintegration step of viral particle assembly (reviewed in [54]). Among the PIC constituents, IN is a logical and probable candidate for facilitating the efficient nuclear import of cDNA, since it has karyophilic properties [55-61]. Reflecting the pleiotropic activities of IN, non-replicative IN mutants of HIV were divided in two phenotypic classes depending on their defects [54]. The properties of IN mutants of PFV are less extensively described, and we suspected that PFV IN could play a key role in early preintegrative steps.

In an attempt to better characterize the properties and substrates of the original IN of PFV, we analyzed both its *in vivo *properties and *in vitro *activity. We observed that the 2-LTR circles could serve as templates for the 3' processing reaction of the IN. This allows spumaretrovirus to follow a symmetrical mechanism of integration  and leads to reexamine the role of 2-LTR molecules and the importance of preintegrative function of IN.

## Results and discussion

### The mutations inPFV IN do not alter its karyophilic property

Retroviral INs from oncoviruses [62,63], lentiviruses [55,59,64,65] and spumavirus [66] are karyophilic proteins, since they localize to cell nuclei in the absence of any other viral protein. Nuclear accumulation of INs may be a general feature of retroviruses. The intrinsic karyophilic property of retrovirus INs could be of high importance for the import of preintegration complex containing viral genomes in the nucleus (for a review, see [53]), where the transcription step occurs.

**Figure 1:**
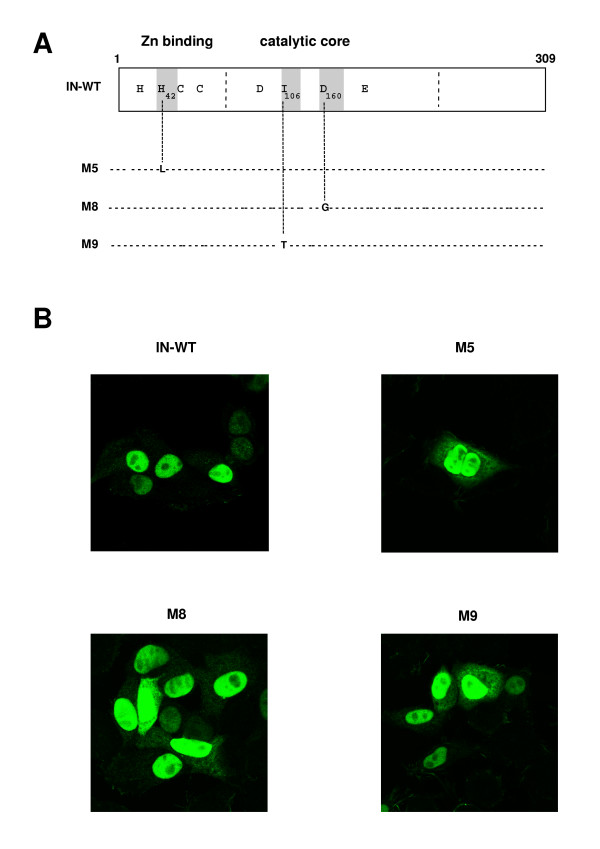
***The mutations in PFV-1 IN do not alter its karyophilic property*. (A) **Schematic representation of foamy virus IN showing conserved motifs and residues between retroviral INs (IN-WT). Critical amino acid residues were mutated as indicated: M5 was mutated within the HH-CC zinc finger domain. In the M8 virus, Asp^160 ^in the invariant conserved catalytic triad, was changed to a glycine residue. Such a mutation has been shown to impair PFV-1 replication [24], likely due to a defective catalytic activity of the protein, as reported in HIV [50]. Another mutation was introduced at Ile^106 ^in the M9 mutant, since this mutation had been described to abolish the *in vitro *integration activity of the protein [24, 27]. **(B) **Confocal microscopy analysis of WT PFV-1 IN and of mutants M5, M8, M9 IN. HeLa cells were transfected with plasmids expressing the WT or mutant IN, fused to the Flag epitope. After 36 hours, cells were fixed, permeabilized, and stained with anti-Flag-antibodies. Series of optical sections at 0.7-μm intervals were recorded. One representative medial section of the immunofluorescence staining is shown.

The 39-kDa PFV virus IN [67] shares significant homologies with other retroviral INs including an amino-terminal HHCC zinc finger, a D, D_35_, E typical active site, and a DNA binding domain (Figure 1A) [68-70]. Three PFV-1 constructs with point mutations at conserved residues of IN were generated: (1) a His^42^Leu mutation within the HH-CC zinc finger domain that has been suggested to be involved in DNA binding (mutant M5, Figure 1A). (2) an Ile^106^Thr mutation which had been described to abolish the *in vitro *integration activity of the protein due essentially to a strong defect in strand transfer, the 3'processing reaction being carried out with an efficacy of 35% compared to the WT IN (mutant M9) [24] and; (3) an Asp^160^Gly mutation (mutant M8) in the invariant catalytic triad which has been shown to impair PFV replication [24], likely due to a defective catalytic activity of the protein, as reported for HIV [69]. As expected, by using a vector encoding PFV-1 IN fused to the Flag epitope, we confirmed that PFV-1 WT IN shares the karyophilic properties as other retroviral IN. PFV-1 IN expressed in Hela-transfected cells was indeed confined to the cell nucleus as detected by immununofluorescence staining (figure 1B). We then evaluated the effects of the IN mutations onto the ability of IN to spontaneously localize into cell nucleus. None of the mutations we introduced did affect the nuclear accumulation of the protein (figure 1B) indicating that these mutations do not affect the ability of IN to be retained in the nucleus by tethering the chromosomes and/or the karyophilic character of IN. We conclude that the IN mutant phenotypes did not result from altered IN cellular localization.

### PFV harboring mutant IN genes are impaired in their replication at an early step

**Figure 2:**
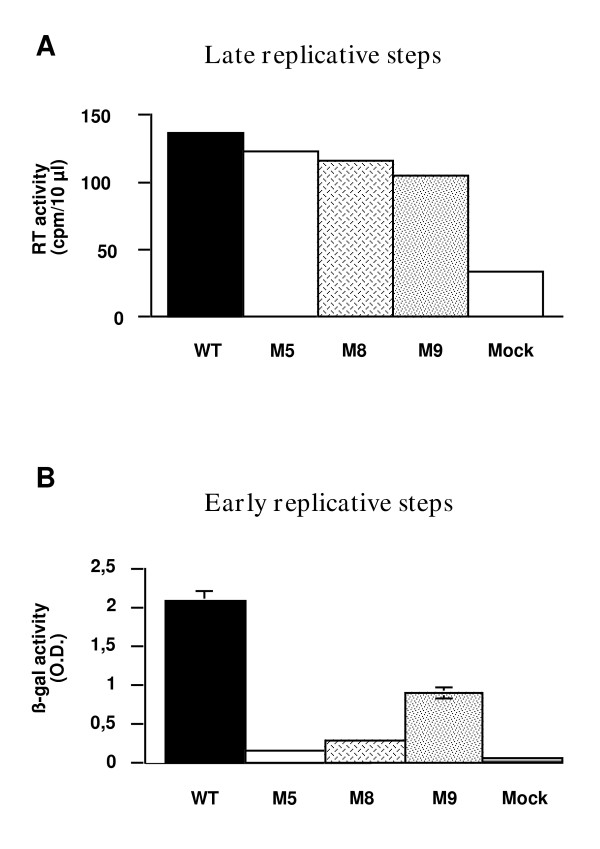
***Impact of the IN mutations on viral replication*. (A) **The late replicative steps – from viral transcription until the release of new virions in the cell supernatant- were studied by determining the reverse transcriptase (RT) activity in the culture supernatant of FAB cells transfected with equal quantities of the various proviral molecular clones. **(B) **To study the early replicative steps, viral infectivity was determined in a single-cycle replication assay using FAB-indicator cells [75]. Cells were exposed to equal amounts of wild-type or IN-mutated viruses for 24 hours, as determined by RT-activity measurements in viral supernatants. Infections were assessed by measuring β-galactosidase activity in cell extracts. Data represent the mean of triplicate infections (+/- SD).

In order to study the impact of IN mutations in the viral context, the three mutations were introduced in the viral molecular clone PFV-1. We first analyzed overall infectivities in situations allowing the dissociation between early and late stages of viral replication. After transfection in FAB cells, transient viral production was found to be similar for both wild type parental and mutant viruses, as measured by reverse transcriptase activity in culture supernatants (Figure 2A). In these cells, only the late phase of virus replication is required to produce virions as transfection allows processes related to the synthesis of viral DNA to be bypassed. Certain point mutations in MLV or HIV IN were indeed described to impair the late replication steps such as virion assembly, production or maturation (viruses classified as class II IN mutant) [38,52,71-74]. This suggested that none of the mutations affected any of the late viral replicative steps, from viral transcription to the release of viral particles (Figure 2A). The impact of IN mutations on viral infectivity was further evaluated in a one-round infection assay based on indicator FAB cells [75]. This assay requires *de novo *synthesis of the viral Tas protein that trans-activates an integrated β-galactosidase reporter gene under the control of PFV LTR in the indicator cells. All mutations were found to affect viral replication in this assay, as well as in multiple-cycle assays in human glioblastoma U373-MG or Baby Hamster Kidney (BHK-21) cells (not shown). Since the DNA transfection experiments demonstrated that viral transcription itself was not affected by the IN mutations, the inability of these mutants to induce expression of the virus trans-activation dependent reporter gene (Figure 2B) indicates that their replication is impaired at an early step, between virus entry and transcription. Of importance, the M9 virus retained nearly 50% of the replication ability of its wild-type counterpart, which was striking in view of the reported inability of IN mutated at this site to integrate DNA mimicking PFV-1 LTR ends *in vitro *[27]. These data confirm that IN integrity is required for PFV replication. As for other retroviruses, it participates at an early pre-transcriptional stage of the replication cycle. Interestingly, it appeared that PFV can still replicate with an IN that has lost its *in vitro *strand transfer activity. Similar paradoxical observations have already been reported for HIV [39,51,76].

### PFV-1 replication defective IN mutants display an abnormal pattern of viral DNA synthesis with an accumulation of 2-LTR circles

To further document the early steps at which the replication of defective mutant IN viruses is impaired, detailed kinetic analyzes of the different viral DNA forms were conducted in infected cells. The importance of IN in the virus replication might be very early since it participates to reverse transcription [23,46-52], and may be even in close contact with the viral DNA all along its synthesis since it was shown to directly interact with the RT [46,47].

**Figure 3:**
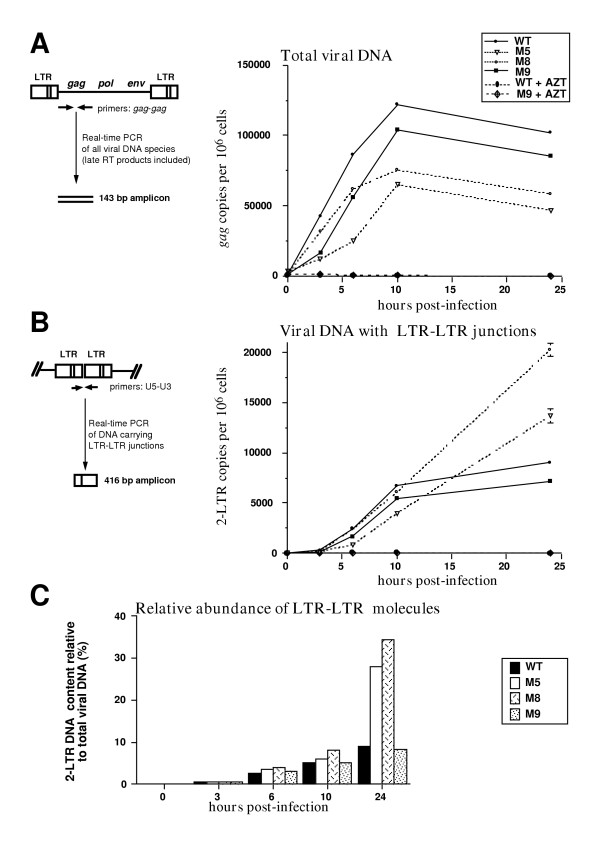
***Decreased viral DNA production by IN-defective viruses is concomitant with an abnormal accumulation of LTR-LTR junctions***. Quantification of viral DNA synthesis was carried out by real-time PCR amplification of total DNA extracts from U373-MG infected cells (equal virion levels as measured by reverse transcriptase activity), collected 3, 6, 10, and 24 hours post-infection. An m.o.i. of 1 for the WT infection as determined by the FAB assay was used. Data are presented for 10^6 ^cells as measured by quantification of the nuclear β-globin gene and standard deviations representing variations between two quantifications of the same sample are given. To ensure that only freshly synthesized DNA, and not contaminating DNA contained in the viral particles input, was analyzed, all infections were performed in parallel control experiments under AZT treatment that inhibits viral neosynthesis. Representative kinetics from 4 independent experiments is presented. **(A) **Total viral DNA was detected using primers allowing amplification of the region of the PFV cDNA at the 5' end of the *gag *gene [40]. **(B) **Viral DNA with 2-LTR junctions was measured using primers that cross the junction between the two LTRs as previously described [40]. **(C) **The abundance of 2-LTR molecules is expressed as the percentage of 2-LTR copies relative to the total viral DNA (*gag*) at each infection time-point.

U373-MG cells were exposed to equal amounts of viral particles. At various time-points after infection, DNA was extracted from infected cells and analysed for total viral DNA content by real-time PCR amplifying a *gag *region. This PCR reaction amplifies all complete reverse transcription products. As shown in Figure 3A, all IN-defective viruses produced viral DNAs containing *gag *sequences indicating that their reverse transcription proceeded through both strand transfers. This DNA represented newly synthesized molecules since the RT-inhibitor AZT abolished DNA production (Figure 3A). However, the amount of viral DNA accumulating in cells infected with M5 and M8 mutant viruses was reduced, as compared to the DNA contents in wild-type virus-infected cells. After 24 hours of infection, viral DNA production increases in cells infected with wild-type or M9 virus (data not shown), likely reflecting new viral cycles which only take place under conditions of productive infection. These data indicate that M5 and M8 IN mutations affect reverse transcription, an IN mutant phenotype also observed in other retroviruses [38,50,51,61].

Various DNA extracts were then analyzed for their content in molecules carrying 2-LTR junctions. As previously shown [40], viral DNA containing a LTR-LTR junction could be detected as early as 3 hours post-infection, and it continuously increased during viral replication (Figure 3B). The kinetics of production of 2-LTR species for IN mutant viruses paralleled that of the wild-type virus, indicating that their reverse transcription products were quite compatible with the formation of viral DNA containing LTR-LTR junctions. Using these quantitative data, we calculated the ratio of 2-LTR *versus gag *containing DNA in the same extracts. As for other retroviruses [77,78], viral DNA species with an LTR-LTR junction represented a minority of the total viral DNA, from 0.6% early in the replicative cycle to a maximum of 9% 24-hour post-infection, in the case of wild-type virus (Figure 3C).

Interestingly, for all IN-mutant viruses, we noticed a marked increase in the proportion of 2-LTR species as compared to the wild-type virus. The over-representation of 2-LTR molecules increased all along infection, reaching a remarkable 35% of total viral DNA in the case of the M8 mutant (Figure 3C). 2-LTR PCR does not allow to distinguish between 2-LTR circles and other molecules containing a LTR-LTR junction such as concatemeric linear or circular genomes. As the later molecules were not described, we assume that the 2-LTR junctions we quantified are indeed carried by circular genomes as in other retroviruses. However, such circles were difficult to detect during spumavirus infection by Southern blot [79], and further studies will be required to precisely answer this question.

Our kinetic analyses revealed that the impaired global production of viral DNA due to inactivation of IN was associated with an abnormal accumulation of 2-LTR DNA species. Importantly, this overaccumulation of 2-LTR species has also been associated with IN-defective HIV viruses [50,80-82]. To explain this observation, it is currently assumed that linear HIV DNA, representing the precursor of integration [3,5], accumulates because it cannot be integrated and is rerouted into the circularization pathway producing 2-LTR molecules in the nucleus [29,83-85]. However, 2-LTR circles are also detected in WT infected cells. In this case, 2-LTR formation was suggested to result from aberrant *att *sequences preventing their recognition by IN [83]. Moreover, since 2-LTR molecules have been detected both in the cytoplasm and the nucleus of PFV WT infected cells [40], as well as at very early time-points in cytoplasm of MLV infected cells [41], overproduction of 2-LTR DNA cannot simply be explained by such a rerouting of non-integrated viral DNA. Alternatively, PFV-1 IN might be directly involved in the processing and/or turnover of viral DNA containing LTR-LTR junctions explaining their accumulation when IN is defective. To address this hypothesis, we tested whether PFV-1 IN might use LTR-LTR circle as a substrate *in vitro*.

### PFV IN can specifically cleave the conserved palindromic sequence found at LTR-LTR junctions to generate 3'-end processed LTRs

**Figure 4:**
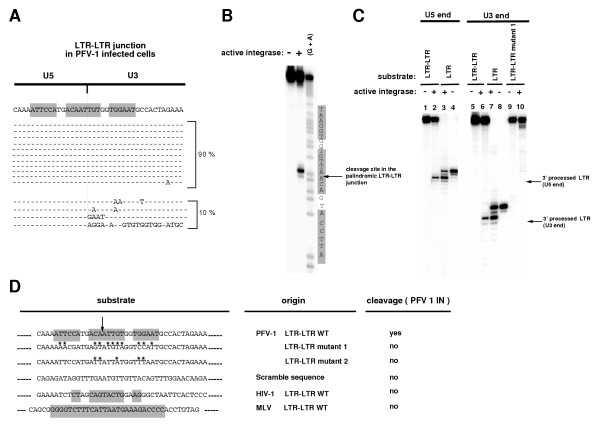
***PFV-1 IN specifically cleaves the conserved palindromic sequence found at LTR-LTR junctions*. (A) **The LTR-LTR junction in infected cells forms a 20 nucleotide-long bipartite palindrome. The LTR-LTR viral DNAs were PCR-amplified, cloned and sequenced following 5-days infection of BHK-21 cells with wild type virus. The vast majority of sequences (90%) were similar whereas approximately 10% had some divergence of the U3 junction. **(B) **The LTR-LTR junction is cleaved by recombinant PFV IN. This purified IN was shown to be functional by its 3' processing activity on the blunt-ends of PFV LTR (see lanes 3 and 7, panel C) and its strand transfer activity (not shown). The U5 strand of an oligonucleotide spanning over the WT LTR-LTR palindromic junction was labelled at its 5' extremity, annealed to its U3 complementary strand and incubated in the presence of PFV-1 IN. Products were resolved on a 15% denaturing polyacrylamide gel. A G+A chemical sequencing reaction was run alongside to identify the cleavage site. A specific cleavage immediately downstream of the conserved 5'CA was obtained. The complementary strand was used for the U3 LTR-LTR junction. (**C) **The cleavage of the LTR-LTR junction by IN is operating on the two strands of the palindrome leading to cohesive digestion fragments (lanes 2 and 6) indistinguishable from the products generated by the classical 3' processing *in vitro *reaction on the blunt-ended LTRs (lanes 3 and 7). Cleavage products were obtained as for panel B. 3' processing of either U5 or U3 blunt double-stranded LTRs was carried out under similar conditions and products were run alongside to confirm the structure of the palindrome cleavage products. Lanes 2, 3, 6, 7 and 10: 150 nM PFV-1 IN; Lanes 1, 4, 5, 8 and 9: 150 nM IN + 20 mM EDTA. EDTA was used to impair the cation-dependant activity of IN. This digestion is highly specific of the viral palindromic sequence since a mutated palindrome (which sequence is indicated panel D) was not cleaved by IN (lane 10). **(D) **A palindrome motif is required for cleavage by PFV-1 IN. Cleavage of oligonucleotides with mutations that disrupt the palindrome motif (mutated nucleotides different from the PFV wild-type sequence are marked with an asterisk), and with a scrambled sequence was assessed. Oligonucleotides carrying different palindromes chosen because they correspond to LTR-LTR junctions of other retroviruses such as HIV-1 and MLV were also tested as putative substrates of the PFV-1 IN. Assays were performed under the same conditions as in Fig. 3C. The ability of the IN to cleave the oligonucleotides onto their two strands is indicated in the right column. The vertical arrow indicates the cleavage site of the wild-type PFV LTR-LTR junction. These experiments were found reproducible in four independent assays.

Sequences located at each end of linear proviral DNA, that are essential for recognition by IN, define the viral attachment (*att*) site. We analyzed sequences connecting the LTRs in the 2-LTR viral DNAs produced in infected cells. We found that these sequences bear a long palindrome composed of a central 8-base motif, flanked on each side by another 12-base palindrome separated from the central one by a 2-nucleotide insertion (Figure 4A). This 20 nucleotide-long bipartite palindrome was highly conserved in 36/40 of the sequenced clones as well as in U373-MG-infected cells, and corresponded to the juxtaposition of blunted 5'-LTR and 3'-LTR ends [24]. Palindromic sequences at the LTR-LTR junctions of the 2-LTR circles were also described in ASV and HIV-1 infected cells, each of them having its unique and specific palindrome (Figure 4D) [29,31].

Since inactivation of PFV IN led to the accumulation of 2-LTR viral DNA containing a palindrome reminiscent of enzymatic restriction sites, we tested whether this palindrome was a possible substrate for the endonuclease activity of IN, as proposed for avian retroviruses [86]. Recombinant PFV IN was produced in E. coli and purified on nickel column. The purified IN, able to catalyze integration *in vitro*, was incubated with a double stranded ^32^P-labeled oligonucleotide containing the palindrome. Reaction products were analyzed by electrophoresis in a polyacrylamide sequencing gel. A cleavage product appeared in the presence of IN confirming that IN harbors endonuclease activity. Moreover, the digestion fragment was found to be unique (Figure 4B and 4C, lanes 2 and 6) and corresponded to a cut between the two consecutive adenines in the middle of the palindrome, as determined by comigration of the sequencing reaction (Figure 4B, lane (G+A)). This digestion was dependent on IN activity as only the initial oligonucleotide was detected when IN was inactivated by EDTA treatment (Figure 4B and 4C, lanes 1 and 5). Moreover, this activity of PFV-1 IN was highly dependent on the target sequence since oligonucleotides carrying mutations that disrupt the palindromic character of the LTR-LTR junction (Figure 4C lane 10 and Figure 4D), and an irrelevant scrambled oligonucleotide (Figure 4D) did not undergo specific cleavage. Finally, PFV-1 IN did not cleave palindromes that are found at HIV-1 and MLV retroviral LTR-LTR junctions (Figure 4D). These data demonstrated that IN double-stranded DNA cleavage activity is restricted to the palindrome at the LTR-LTR junction found in corresponding infected cells and thus carries the same sequence specificity as already documented for the 3'processing of LTR extremities [26]. Detailed analysis indicated that the digestion had operated on the two strands (U5- and U3-end labeling) of the oligonucleotide substrate generating cohesive ends with a 5'-protuding AT (compare lanes 2 and 3, or 6 and 7, Figure 4C).

Altogether, these data reveal a new substrate for IN endonuclease activity. This endonucleolytic activity is able to cleave specifically the palindromic sequence generated at the LTR-LTR junctions of viral DNA. The cleavage of 2-LTR circles into linear genomes justifies revisiting them as functional intermediates in the retroviral cycle. This is reinforced by recent observations showing their stability and contribution to the viral transcription [36,37,77,78]. Interestingly, many DNA viruses replicate by using circular intermediates resembling the retroviral 2-LTR circles, and require the activity of a virally encoded endonuclease reminiscent of the IN. Identification of new IN activity should improve our understanding of the early steps of the retroviral replication cycle, allow screening of anti-retroviral drugs as well as design of new non-integrating retroviral vectors.

### That IN operates on 2-LTR molecules to produce linear DNA with each LTR end 3'-processed avoids the need for asymmetrical integration in spumavirus

**Figure 5:**
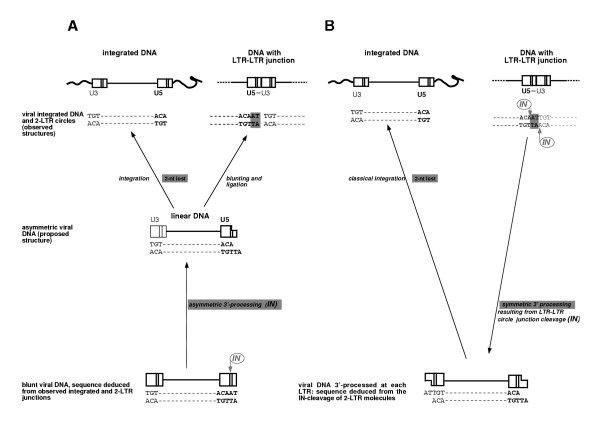
***Asymmetric integration is not required to understand the sequences of integrated and 2-LTR molecules observed in PFV-1 infected cells*. (A) **The asymmetric integration in PFV-1 virus was proposed to account for the sequences of both integrated and 2-LTR viral molecules as observed in the infected cells [24, 25]. This unusual proposed integration was able to solve the problematic lost of only 2 nucleotides between U5 extremity of the integrated molecules and the putative U5 free end, whereas the U3 end remains unchanged. This assertion was based on the following model: the linear substrate for integration is produced by two 3'-processing reactions at each end of a blunt molecule. Of note, such blunt linear molecules have never been detected in infected cells and their structure was deduced from the observed 2-LTR circles sequences. Such deduction is based on the idea that 2-LTR circles result from the ligation of blunt linear DNA. However the actors of this reaction are still unknown. **(B) **We propose a revised version where the PFV-1 integration remains classical. A single reaction of PFV-1 IN onto the palindrome at the LTR-LTR circle junction can generate a linear DNA with its two 3' ends processed. The subsequent integration then eliminates the two nucleotides that are lost between the observed sequences of the LTR-LTR junction and the integrated provirus.

PFV IN was suggested to be unrelated to other retrovirus INs because of its apparent inactivity on the U3 LTR end of linear molecules, and the integration process of spumavirus was proposed to be asymmetrical [24,25]. The asymmetric integration has been deduced from the sequences of both integrated and 2-LTR viral molecules (Figure 5A). The usual replication model supposes that the reverse transcription stage leads to linear DNA with blunt-ends. However, these ends are difficult to detect and sequence. Their structure had been previously deduced from the sequence at the LTR-LTR junctions. Indeed, the latter are themselves supposed to be formed by the intramolecular ligation between the two blunt-ends of linear DNA by an unidentified mechanism. As only two nucleotides are lost during integration, the PFV integration process was proposed to be unusual (figure 5A).

In light of our observation that 2-LTR molecules are possible substrates for PFV-1 IN (Figure 4), the 3'-processing of both ends of the linear DNA might be generated in a single reaction that produces the two 3'-processed ends simultaneously (Figure 5B). Such concerted processing might explain the influence of one LTR on the processing of the other, as observed for HIV-1 [20]. The subsequent integration of such processed extremities would eliminate the two nucleotides that are lost between the LTR-LTR junction and the integrated provirus. No asymmetric integration is required to account for the previous observations [24,25]. This mechanic, when generalized to other retroviruses carrying a different palindrome at the LTR-LTR junction, would result during integration in the loss of the number of nucleotides comprised between the conserved CA.

In support of our symmetrical integration model, Pahl and Flügel [26] previously reported an efficient 3'-processing activity of PFV IN on LTR containing the two additional nucleotides AT. The substrate of concerted processing corresponds to the extended substrate they tested. We confirmed the 3'-processing cleavage of the extended U3 LTR carrying an additional AT (Figure 4C), as well as the fact that the 3'-processing does not occur onto the shorter U3 LTR lacking these nucleotides (not shown).

### Integration depends on preintegrative IN activity

**Figure 6:**
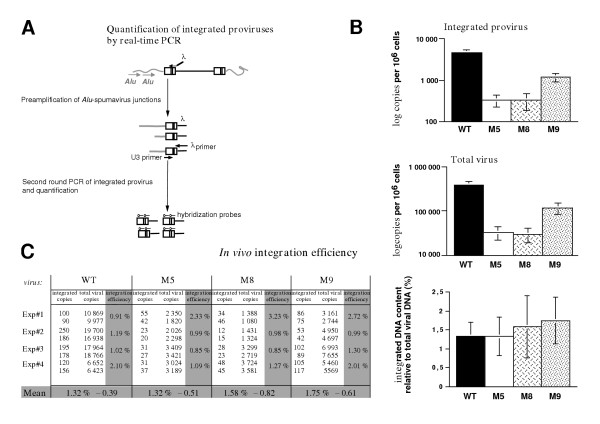
***Integration of IN-defective viruses*. (A) **A quantitative assay based on a real-time RACE-PCR reaction was designed, amplifying Alu-LTR junctions between the cell genome and integrated proviruses twenty-four hours post-infection. PCR amplifications of existing Alu-PFV-1 LTR junctions were subjected to a second quantitative round of real time PCR with PFV-1 LTR-specific primers. Fluorogenic hybridization probes were used to quantify the amplification products. Infected cells with known copy numbers of integrated proviruses were used as quantification standards. The assay is highly sensitive since it allows detecting 25 proviruses copies in 50,000 human cells. Control reactions are detailed in the Material and methods section. **(B) **Detection of integrated viral DNA following infection of IN-mutated viruses. Quantitation of viral DNA accumulated in PFV-1 infected cells was carried out by real-time PCR of total DNA extracts from U373-MG infected cells (m.o.i. of 1) collected at the completion of the first viral replication cycle, 24 hours post-infection. Total viral DNA (*gag *quantifications) and integrated proviruses were quantified in duplicate using real-time PCRs. Data obtained in one representative infection from four independent experiments are expressed as integrated DNA copies per million cells (logarithmic scale) as determined by a human β-globin quantification in cell extracts ("Integrated provirus" panel). Total DNA copies per million cells (logarithmic scale) present in the same extracts are presented in the lower panel. Standard deviations representing variations between two quantifications of the same sample are given. **(C) **Integration efficiency in PFV-1 infected cells. Integration efficiency was determined by normalizing the number of integrated proviruses (mean of duplicates) with the total number of viral DNA molecules (mean of duplicates) present in the same extract. Raw LightCycler data from four independent experiments are presented in the upper table. Mean of integration efficiencies from these four experiments are figured in the lower histogram.

Integration was reported to be a very rare event in spumaviruses [87,88], except in chronically infected cell situations [89]. To document this point in our conditions, we quantified the integration events for PFV-1 WT and IN mutants. To this end, we designed a highly sensitive quantitative real-time RACE-PCR reaction, amplifying Alu-LTR junctions between the cell genome and integrated proviruses (detecting 25 integrated proviruses per 50 000 cells, Figure 6A). U373-MG cells were infected with equivalent amounts of viral particles as measured by RT activity and the quantity of integrated viral molecules was analyzed 24 hours later, a time-point at which the first round of infection is achieved. As shown in Figure 6A, and as expected [87,88], only a small fraction of total wild-type PFV DNA was integrated (range of 0.9–2.1%). The M8 and M9 mutant INs used in our study failed to integrate oligonucleotides mimicking the PFV LTR DNA ends into a target plasmid *in vitro *[26]. We therefore assessed the ability of viruses carrying the same IN mutations to integrate *in vivo*. We could detect integrated DNA after infection with viruses carrying inactive INs (Figure 6B upper panel). However, with the exception of the semi-replicative M9 virus, IN mutants yielded significantly fewer integrated proviruses than the wild-type (Figure 6B). Similar observations have been reported in cells infected with IN-defective HIV and the presence of integrated proviruses was attributed to integrase-independent integration events depending on cell enzymes [81]. Another explanation could rely on the fact that IN mutants produced less linear DNA as a substrate for integration. The altered viral DNA production is likely reflected by the reduced amounts of total viral DNA quantified in the same extracts (Figure 6B lower panel). We compared integration ratios with and without functional IN by normalizing integrated proviruses values with the total number of viral DNA copies present in infected cells. Strikingly, the percentage of integrated DNA was not modified by the presence of a defective IN (Figure 6C). Thus, the level of integrated provirus depends on the global viral DNA pool available in the infected cells. And such global viral DNA content itself depends on the early activity of the viral IN as shown above.

### Role of IN in PFV retrovirus replication cycle

We conclude from these experiments that PFV IN displays a specific activity on the 2-LTR circles, which may constitute a substrate for the 3'processing reaction *in vivo*. This action of IN generates linear DNA that might be then integrated in the cell genome following a classical symmetrical integration process. The fact that early actions of IN may influence later steps of replication, including integration, certainly participates in the pleiotropic effects of IN mutations. Finally, IN seems to be essential not because of its participation to the integration *per se *but for its upstream activities able to influence integration efficacy.

**Figure 7:**
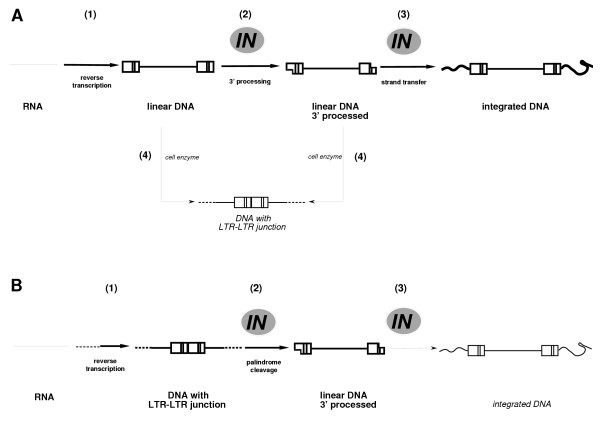
***Role of IN in retrovirus replication cycle*. (A) **Classical model of early steps in retrovirus replication. IN plays a role in the 3' processing as well as in the integration itself, these two steps being separated both in time and in space. Following synthesis of linear blunt-ended DNA in the cytoplasm (step 1 in Fig. 7A), IN cleaves their 3' termini, thus eliminating the terminal two bases from each 3'end (step 2). The resulting recessed 3'OH groups provide the attachment sites of the provirus to host DNA, an attachment which is performed only after import of 3'processed DNA into the nucleus where the final step of the integration process occurs (step 3). Circular DNA carrying LTR-LTR junctions are reportedly formed from linear DNA *via *the action of cellular ligases (step 4). The circularization is considered to be an alternate fate of linear DNA that has not integrated, and may indirectly explain why DNA bearing LTR-LTR junctions accumulates to high levels in cells harboring integration-defective viruses. This classical model considers that functions of IN in processes other than integration are secondary. **(B) **Alternate retrovirus replication model. IN cleaves the LTR-LTR junction generated at the reverse transcription step (step 1) to produce 3'end-processed linear DNA (step 2). This specific activity of the IN explains the pleiotropic effects of this protein and the phenotypes associated with its mutagenesis. First, since linear DNA is the direct product of a reaction that is catalyzed by IN, its levels would decrease under IN-defective conditions. Moreover if LTR-LTR junction molecules indeed constitute the substrate for IN, their amount would increase as a direct consequence of defective IN. Second, decreased levels of integrated proviruses would be an indirect result of the decreased pool of 3'processed IN-catalyzed linear DNA molecules that are available for integration (step 3). In this model, 2-LTR molecules are a replication-intermediate. Low levels of these molecules would be due to their rapid processing by IN in the wild-type infections. Rapid processing might also explain the presence of linear molecules with 3' processed ends in the cell cytoplasm during diverse retroviral infections, even though no blunt-ended linear molecules can be recovered from infected cells. Thus, apart from participating in retroviral DNA integration *per se*, IN would act upstream by controlling linear DNA production. This function of IN, as included in the modified replication model presented here, provides a parsimonious interpretation of the pleiotropic effects observed in cells infected with IN mutants.

Our findings that a loss of endonuclease IN activity results in both LTR-LTR accumulation and an associated reduction in viral DNA production leads us to propose a direct role for retroviral integrase in the production of viral DNA. Thus, a modified replication model is presented in Fig. 7B. It is accepted that the encounter between viral DNA and IN occurs very shortly after viral DNA synthesis, since cytoplasmic viral DNA is mostly found as linear molecules with 3' processed ends resulting from IN endonucleolytic action in the cytoplasm [13-15]. In our model, DNA molecules containing LTR-LTR junction would be generated during the reverse transcription process and cleaved rapidly by the IN, leading to the production of linear DNA harboring 3'-processed ends. This would account for the rarity of linear DNA with blunt ends in the cytoplasm of infected cell, as well as for the presence of 2-LTR circles in the cytoplasm of retrovirus infected cells at early times post infection [40,41]. Additionally, it would explain the data from *att *site mutagenesis experiments showing that mutation of one LTR precludes the processing of the other LTR [20]. These results were initially interpreted to represent a concerted activity of IN on the two viral LTRs ends that must be simultaneously cleaved in infected cells. In view of our results, these data might be understood as resulting from the endonucleolytic activity of IN on palindromic LTR-LTR junctions. Such processed DNA could then undergo integration. In this interpretation, a unique endonucleolytic action of IN at an early step would explain many of the phenotypes associated with IN mutations, including the increasing abundance of 2-LTR molecules at the expense of linear and integrated DNA in IN-defective viruses. It underlines that *in vivo *integration is performed in two steps that are uncoupled both in time and in space, *ie *3' processing in the cytoplasm and integration *per se *in the nucleus. It also illustrates why and how certain *in vitro *integration-defective viruses such as our M9 mutant or HIV mutants [39,51,76] are still replicative. The IN activity demonstrated in this report allows processing the circles – currently considered as dead-end molecules- into the replication pathway. Additional support to this conclusion is present in the HIV literature where episomal circular DNA were shown to turn over by degradation rather than through death or tissue redistribution of the infected cell itself in HIV-1 infected individuals [42]. Finally, our data imply that circular retroviral genomes are fully functional replication intermediates, first as substrates for transcription and second as precursors of linear unintegrated DNA.

Although the consensus sequences in the C ter region of IN may differ between the lentiviruses and the nonlentiviruses, the carboxyterminal region of IN is well conserved in all retroviruses [80], and further studies are now required to evaluate whether the revised replication model we propose here, applies to all retroviruses. The fact that the typical phenotype associated with a defective IN, either due to mutations or inhibitors, resulting in reduced DNA synthesis but a persistence of integration and an accumulation of 2-LTR molecules, is commonly observed among retroviruses [73,82,90], argues in favour of a conserved IN function. Such an early participation of IN sheds new light on reports showing both that viral transcription occurs from nonintegrated HIV DNA [38,44,45,91], and that the most prevalent form of HIV DNA during the asymptomatic phase of infection is full-length unintegrated DNA [42,92]. Whereas IN activity is clearly required, formation of integrated provirus as an obligate step of retroviral replication now needs to be reconsidered. On the other hand, early preintegrative activities of IN are of capital importance. This provides new answers to the puzzling question of why is integration essential to retrovirus replication, when many authors have shown that unintegrated genomes are abundant and expressed [36-39,42-45,93]. Our proposal is simply: integrase is essential, integration is not; and IN is required given its critical preintegrative influence on genomic DNA production *in vivo*, as we precisely measured here.

Given the above, retroviruses better fit the classical schemes of distinct lytic and lysogenic phases exemplified by the lambda phage: integration (lysogeny) contributes to viral persistence and pathogenesis, but it is not essential for acute viral production (lytic cycle). Finally, a fascinating evolutionary conservation appears between retroviruses and DNA viruses (such as poxviruses). All use circular DNA intermediates and a specialized endonuclease activity for genome production.

## Methods

### Cells, virus infections and reagents

BHK-21, FAB, HeLa and U373-MG cells were cultivated in DMEM with 10% foetal calf serum, 1 μg per ml of streptomycine-streptavidine. For FAB indicator cells, 1 μg per ml of G418 (Sigma) was added.

PFV-1 virus stocks were prepared by transfecting BHK-21 cells with the PFV-1 molecular WT and mutant clones using the calcium phosphate method. Cells were infected by WT and mutant viruses with same amounts of viral particles, as evaluated by a reverse transcription assay. The culture medium was changed two hours post-infection with fresh medium.

Cell free virus stocks were titrated on FAB cells [75]. In some experiments, infected cells were treated with 3'-azido-3'-deoxythymidine (AZT, Sigma) at 100 μM.

### DNA quantifications by real time PCR

Total DNAs were extracted from 10^6 ^cells using the DNA Blood Mini kit (Qiagen) in a final volume of 200 μl and analysed by real time PCR as described previously [40]. Integrated viral DNA was also quantified by two rounds of PCR [94]. The first one amplifies integrated DNA using primers ALU1 (5'-CCT CAG CCT CCC GAG TAG CTG GGA-3'), ALU2 (5'-CTG TAA TCC CAG CAC TTT GGG AGG C-3'), and λ TSPA (5'-**ATG CCA CGT AAG CGA AAC T**TA GTA TAA TCA TTT CCG CTT TCG-3'). Sequence in bold represents a sequence in the lambda phage, which is unknown in all mammals' databanks. The other part of the sequence of λ TSPA primer can hybridize in PFV LTR. Amplification was performed in a 20 μl reaction volume containing 1X Light Cycler Fast Start DNA Hybridation probes, 3.5 mM MgCL_2_, 300 nM of primer ALU1, ALU2 and 10 nM of primer λ TSPA. The same mix, containing only primer λ TSPA, was prepared. DNA from U373-MG chronically infected cells was used as a standard for integrated copies. All reactions were further diluted in a final volume of 200 μl of water. 2 μl over 200 μl was used for the second PCR. This amplification was performed with 300 nM of each primers Nested R (5'-GAA ACT AGG GAA AAC TAG G-3'), lambdaT (5'-ATG CCA CGT AAG CGA AAC T-3') and 100 nM of each hybridation probes SpuFL (5'-CAC TCT CGA CGC AGC GAG TAG TGA A X-3') and SpuLC (5'-GCC TCC CGT ACA ATC TAG AAA CTA TCC T p-3'). This assay is quite specific of integrated provirus only, as attested by performing the following control reactions: – a carry-over control in which all primers were omitted in the first PCR, data obtained indicated always that the second-round amplification of nonpreamplified viral DNA is efficiently prevented; -a parallel reaction with the *Alu *primers in the first-round PCR, in order to calculate the linear amplifications resulting from all the viral DNA species. The copy number due to the linear amplification was systematically subtracted from the signal obtained in the presence of *Alu *primer. We evaluated that this interfering amplification never exceeded 6.7 % of the global amplification.

Quantifications were performed with the LightCycler software Version 3.5 according to manufacturer's instructions.

### Virion-associated RT assays

48 hours post transfection viral supernatants were collected. 10 μl of viral supernatant was incubated with 20 μl of reaction buffer (Tris pH 8 50 mM – KCl 75 mM – Dithiotreitol 2 mM – rA/dT 25 μg/ml – NP40 0,05% – MnCl_2 _5 mM – dTTP α-^32^P 20 μCi/ml). The reaction mixtures were incubated at 37°C for 90 min. 10 μl of the reaction was spotted onto DE81 filter and allowed to dry. The filters were washed four times with 2xSSC (1xSSC is 0.15 M NaCl plus 0.015 M sodium citrate) for 5 min each, followed by two washes with 95% ethanol. The filters were then dried and counting by scintillation fluid.

### Construction of Flag-PFV IN mutants and their cell localisation by immunofluorescence staining

To express the INs in the absence of other viral products, we used the pFlag expression vector [95]; in which we inserted the PFV-1 IN sequence under the control of the simian virus 40 promoter. The IN fragment was amplified by PCR with the following primers, which created a *BamH1 *and an *XhoI *restriction site at the 5' and 3' ends, respectively, of the IN sequence: 5'-GGA TCC TAC ATA TTT TTT AGA AGA TGG C-3'; and 5'-CTC GAG TTA TTC ATT TTT TTC CAA TGA TCC-3'. The resulting PCR fragment was digested with *BamHI *and *XhoI *and ligated into the corresponding cloning sites of pSG-Flag [95], in the plasmid called pSG-FlagIN PFV. The pSG-FlagIN PFV expression vector was used for the mutagenesis, with the Quick Change mutagenesis kit (Stratagene), and the primers: 5'-CAA TTT GGC TCT CAC AGG ACG TGA AGC C-3' and 5'-GGC TTC ACG TCC TGT GAG AGC CAA ATT G-3' for the M5 mutant; 5'-ATT CAC TCT GGT CAA GGT GCA GC-3' and 5'-GCT GCA CCT TGA CCA GAG TGA AT-3' for the M8 mutant; and 5'-GGC AAA GGG CCA GTA TAG TCA AT-3' and 5'-ATT GAC TAT ACT GGC CCT TTG CC-3' for the M9 mutant.

HeLa cells (2 × 10^5^) were spread on glass coverslips in 24-well plates, transfected with 1 μg of the corresponding plasmids, and stained for immunofluorescence 36 hours later. Cells were fixed in 3.7% formaldehyde-PBS for 20 min, washed three times in PBS, and incubated for 10 min in 50 mM NH_4_Cl to quench free aldehydes. Cells were washed three times in PBS and incubated in a permeabilization buffer (0.05% saponin, 0.01% Triton X-100, 2% bovine serum albumin, PBS) for 15 min and incubated 1 h with the first MAb (M2 anti-Flag MAb at 7.5 μg/ml) in permeabilization buffer. Cells were washed three times in permeabilization buffer and incubated with Cy3-conjugated anti-mouse MAbs (Amersham) at a final dilution of 1:200. Cells were washed three times in permeabilization buffer and once in PBS and mounted in 133 mg of Mowiol (Hoechst) per ml-33% glycerol-133 mM Tris HCl (pH 8.5). Confocal microscopy was performed and optical sections were recorded. One representative medial section was mounted by using Adobe Photoshop software.

### Construction of PFV proviruses

We inserted a DNA fragment containing the PFV-1 IN sequence into a Litmus 38 plasmid, in which a *PacI *site had been added. The viral fragment was amplified by PCR with the following primers: 5'-GGA TCC TAC ATA TTT TTT AGA AGA TGG C-3' and 5'-CTC GAG TTA TTC ATT TTT TTC CAA TGA TCC-3', and cloned after a *BspEI-PacI *digestion into the modified Litmus. This plasmid containing the WT IN was used for the mutagenesis, with the Quick Change mutagenesis kit and the primers used above for the expression IN vector mutagenesis. After the mutagenesis, the *PacI-BspEI *digestion fragments from the mutated Litmus vectors were substituted for the corresponding sequence of the PFV-1 full-length clone. All constructions were confirmed by DNA sequencing of the entire PCR-amplified fragment.

### 2 LTR junction sequence analysis

Total DNA from acutely BHK-21 infected cells of two independent infections were extracted and analyzed by a PCR amplification specific for the LTR-LTR junction from the 2-LTR circles, using the following primers: R, 5'-TAC GAG ACT CTC CAG GTT TG-3'; and U3, 5'-CGA CGC AGC GAG TAG TGA AG-3' and the Pfu polymerase (Stratagene) [40]. PCR products were cloned in a pSK+ plasmid (PCR-Script cloning kit, Stratagene). 50 independent cloned were sequenced.

### Construction and purification of PFV recombinant IN

Histidine-tagged PFV-1 IN, corresponding to aminoacids 752-1143 of the Pol polyprotein, was expressed and purified by nickel affinity. The preparation and purification of recombinant PFV-1 IN protein were performed as described for HIV IN [96]. To obtain wild type IN protein, plasmid pET15b (Novagen) was digested with *NdeI *and *BamHI*. The DNA fragment containing the PFV IN was obtained from pHSRV clone C55 by PCR using the *Pfu *DNA polymerase (Stratagene). The sequence of the primers used to amplify the fragment were 5'-ACA TAT GTG TAA TAC CAA AAA ACC AAA CCT GG-3' and 5'-AGG ATC CTT ACT CGA GTT CAT TTT TTT C-3'. PCR amplifications were done at 92°C for 1 min, 55°C for 45 s, and at 72°C for 90 s; the cycle was repeated 28 times. The resulting PCR fragment were digested with *NdeI *and *BamHI *and ligated into the corresponding cloning sites of pET15b. Plasmid pET15bIN was used to express the His-tagged IN in *E. coli *BL21 (DE3) cells. 500 ml of BL21 (DE3) pET15bIN cells was grown at 37°C in LB medium (supplemented with 50 mg/ml ampicilin) to an A^600 ^of 0.6–0.8. To induce IN protein expression, isopropyl-1-thio-β-D-galactopyranoside was added to a final concentration of 1 mM; bacteria were grown for another 4 hours and harvested by low speed centrifugation. The pellet was resuspended in 24 ml of 50 mM Tris-HCl, pH8, 1 M NaCl, 4 mM β-mercaptoethanol (buffer A). Cells were lysed with French Press and centrifugated at 14,000 rpm and 4°C for 30 min to remove cells debris

The supernatant was filtered (0.45 μm) and incubated over night with Ni-NTA agarose beads (Qiagen). The beads were washed with 10 volumes of buffer A. Then, IN was purified under native conditions according to manufacturer's instructions using batch procedure. His-tagged IN was eluted with buffer A supplemented with 50 μM ZnSO_4 _and 1 M imidazole. The IN concentration was adjusted to 0.1 mg/ml in buffer A and dialysed over night against 20 mM Tris-HCl, pH 8, 1 M NaCl, and 4 mM β-mercaptoethanol. Fractions were aliquoted and rapidly frozen at -80°C.

### Nucleic acid substrates

All oligonuleotides U5B (5'-CCT TAG GAT AAT CAA TAT ACA AAA TTC CAT GAC AAT-3'), (U5A 5'-ATT GTC ATG GAA TTT TGT ATA TTG ATT ATC CTA AGG-3'), U3 B (5'-ATT GTG GTG GAA TGC CAC TAG AAA T-3'), U3A (5'-ATT TCT AGT GGC ATT CCA CCA CAA T-3'), LTR-LTRB (5'-CCT TAG GAT AAT CAA TAT ACA AAA TTC CAT GAC AAT TGT GGT GGA ATG CCA CTA GAA AT-3') and LTR-LTRA (5'-ATT TCT AGT GGC ATT CCA CCA CAA TTG TCA TGG AAT TTT GTA TAT TGA TTA TCC TAA GG-3') were purchased from Eurogentec and further purified on an 15% denaturing acrylamide/urea gel. 100 pmol of U5 B, U3 B and LTR-LTR B were radiolabeled using T4 polynucleotide kinase and 50 μCi of [γ-^32^P]ATP (3000 Ci/mmol) during 2 hours at 37°C. The T4 kinase was heat inactivated, and unincorporated nucleotides were removed using a Sephadex G-10 column (Pharmacia). NaCl was added to a final concentration of 100 mM and complementary unlabeled strand was added to either U5 B, U3 B or LTR-LTR B. The mixture was heated to 90°C for 3 min, and the DNA was annealed by slow cooling.

### LTR processing, LTR-LTR junction cleavage

Processing and LTR-LTR cleavage were performed in buffer containing 50 mM Hepes, 5 mM DTT and 10 mM MgCl_2_. 150 nM of PFV-1 IN was used for reaction. The reaction was initiated by addition of substrate DNA, and the mixture was incubated 2 hours at 37°C and stopped by phenol/chloroform extraction. DNA products were precipitated with ethanol, dissolved in TE containing 7 M urea and electrophoresed on a 15% denaturing acrylamide/urea gel. Gels were analysed using a STORM Molecular Dynamics phosphorimager.

## List of abbreviations

*Att*, attachment site

HIV, human immunodeficiency virus

IN, integrase

LTR, long terminal repeat

PFV, primate foamy virus

PIC, preintegration complex

RT, reverse transcriptase

WT, wild-type

## Authors' contributions

OD carried out all the experiments concerning the phenotype analysis of the viruses in the cell context including constructions, viral kinetics and real-time PCR, and participated to the analysis of the data. CP contributed to the design and coordination of the study, supervised the experimental work, participated in the analysis and interpretation of the data, and drafted figures and the manuscript. HL participated in the acquisition of the biochemical datas and in their interpretation. GM contributed to the acquisition of biochemical datas. JFM contributed and supervised biochemical analysis of integrase *in vitro*. PS conceived the original ideas, designed and coordinated the study, and took part in writing the manuscript. All authors read and approved the final manuscript.
